# Direct Oral Anticoagulants in Dental Patients Including the Frail Elderly Population

**DOI:** 10.3390/dj4010007

**Published:** 2016-03-19

**Authors:** Hui Yin Lim, Prahlad Ho

**Affiliations:** 1 Department of Haematology, Northern Health, 185 Cooper Street, Epping VIC 3076, Australia; prahlad.ho@nh.org.au; 2 Department of Haematology, Austin Health, Studley Road, Heidelberg VIC 3084, Australia

**Keywords:** direct oral anticoagulants, factor Xa inhibitor, direct thrombin inhibitor, bleeding, frail elderly

## Abstract

Direct oral anticoagulants (DOACs) have led to a paradigm shift in the field of anticoagulation, providing safe and convenient anticoagulation without the need for regular blood testing. Currently, there are three major DOACs available—Factor Xa inhibitors (apixaban and rivaroxaban) and direct thrombin inhibitors (dabigatran)—that are available for use in atrial fibrillation and venous thromboembolism. While these agents have been shown to be as effective as warfarin, with a similar or better bleeding profile, there remains some concern of the use of these drugs in vulnerable populations, such as the frail elderly patients; particularly since reversal agents and drug monitoring are not routinely available. We aim to provide a review of the use of DOACs and the impact of DOACs on dental treatment in the elderly population.

## 1. Introduction

The availability and convenience of direct oral anticoagulants (DOACs) have led to their increasing use in the management of venous thromboembolism (VTE) and in stroke prevention in patients with atrial fibrillation (AF). This is particularly important in the older population where there is increased prevalence of AF and VTE [1,2]. The safety and efficacy data in these agents have been well established in multiple large randomised controlled trials (RCT), with proven non-inferiority when compared to vitamin K antagonists (VKA) and improved intracranial bleeding profile in some of the agents [3,4,5,6,7]. However, despite the favourable profile and convenience of these agents, there remain concerns regarding their use in the elderly frail population who are also at higher risk of falls and bleeding complications [8,9]. This is further compounded by the lack of readily available reversal agents, though we acknowledge that antidotes are in development and soon to be approved, which will substantially change the safety profile of DOAC use. Similarly, while we can measure DOAC drug levels and on-therapy plasma levels are well-known, their therapeutic range is unknown and there is no effective way of measuring the *in vivo* anticoagulation effect of these patients [10]. 

Patients on therapy with DOACs requiring dental procedures are becoming increasingly common and the balance between the antithrombotic benefits *versus* the bleeding complication risks needs to be evaluated before the cessation of anticoagulation prior to dental procedures. Several guidelines and recommendations have recently been published in order to address these issues given the heterogeneity in the clinical practice [11,12,13].

We aim to provide a review of the use of DOACs in the elderly population and the current recommendations of management of DOACs in patients requiring dental procedures.

## 2. The Evidence for Direct Oral Anticoagulants (DOAC)

DOACs have been extensively investigated in multiple RCT in both AF and VTE management and they have been shown to be non-inferior to VKA with no increase in stroke risk or VTE recurrence [3,4,5,6,7]. In addition, DOACs have been shown to have lower all-cause mortality (Odds ratio (OR) 0.88; 95% Confidence Interval (CI) 0.82–0.95) and intracranial haemorrhage (OR 0.46; 95% CI 0.33–0.65) compared to VKA, although this may be at the expense of increased rate of gastrointestinal bleeding (OR 1.70; 95% CI 1.47–1.96) with some agents [14,15,16]. The biggest advantage of these agents is the stable pharmacokinetic and pharmacodynamic profiles, which negates the need for regular INR monitoring, hence providing significant benefits and convenience for patients. Moreover, the interindividual variations and erratic peak and trough of INR based warfarin dosing may lead to increased complications such as thrombotic complications with subtherapeutic INR and conversely, increased bleeding when supratherapeutic. Interestingly, despite best efforts, the time in therapeutic range (TTR) of warfarin remains suboptimal at approximately 65%, but may vary substantially in different countries and depending on the presence of anticoagulation clinics [17].

The three most commonly used DOACs are dabigatran, rivaroxaban and apixaban. Table 1 summarises the pharmacologic properties of these three agents.

Dabigatran is a competitive direct thrombin inhibitor with rapid onset, short half-life and relatively fewer drug interactions. It is predominantly cleared via the kidneys (~80%) and hence should be used with caution in patients with moderate renal impairment [4,18]. The thrombin time (TT) is the most sensitive coagulation assays available to detect the presence of dabigatran although it is too sensitive to provide an estimation of drug effect. HEMOCLOT, or dilute thrombin time, has been developed to estimate dabigatran levels, but do not provide an estimation of *in vivo* anticoagulation effect [19,23,24]. The recent US FDA approval of idarucizumab, an antibody fragment, which has been shown to completely reverse the blood anticoagulant effect of dabigatran within minutes with minimal adverse effects, is a major advancement for dabigatran. This facilitates dabigatran reversibility in bleeding patients and those who require urgent procedures, and allays concerns about lack of drug reversibility [21]. However, we note that the impact of these reversal agents on the extravascular compartment, as compared to the better documented intravascular blood compartment, remains unclear. Moreover, in clinical trials, the cost of idarucizumab is estimated to be similar to coagulation factor concentrates used for warfarin reversal, though the actual drug costs in many countries have not been determined and this may impact on usage [25,26]. We also note that idarucizumab is not readily available in all hospitals at present time and the indication of when to use this drug has not been fully elucidated. 

Direct factor Xa inhibitors such as Rivaroxaban and Apixaban bind competitively to the active site of factor Xa and are more dependent on hepatic metabolism. While activated partial thromboplastin time (APTT) is more sensitive to the direct thrombin inhibitors, prothrombin time (PT) is the most sensitive routine coagulation assay for detecting rivaroxaban, though this varies with the PT sensitivity in each laboratory [27]. Conversely, a normal PT and APTT do not exclude the presence of the anticoagulant effect of apixaban [28]. Andexanet alfa is currently undergoing phase III trials [22] with promising preliminary results. It is a recombinant modified human factor Xa decoy protein that targets factor Xa inhibitors with high specificity, thus restoring the activity of the endogenous factor Xa and thus normal haemostatic activity while reducing the levels of anticoagulant activity. These agents will change the landscape of DOACs although it may be a period of time before they are available for clinical use.

The rapid onset and offset of the DOACs also negates the need of “bridging” parenteral anticoagulant in acute thrombosis or during periods where individuals are not on anticoagulation, such as peri-procedural period. This is pertinent given that “bridging” anticoagulation can increase risk of bleeding during the peri-procedural period with no additional benefit especially in patients with atrial fibrillation [29,30,31,32]. 

## 3. Concerns with DOACs

While DOACs do not require routine monitoring, there may be situations that require the determination of residual anticoagulant effect such as bleeding patients and those with borderline renal and liver function. Routine coagulation blood testing, such as PT and APTT, are not reflective of the anticoagulation effect of DOACs [19,23]. Specific tests for the quantitation of drug levels have been developed (Table 1) but are generally not widely available outside major tertiary centres and even when available, the therapeutic range or target for these agents is unknown. While the clinical relevance of the drug levels remains to be elicited, there is increasing consensus that point measurement may be required in some situations like bleeding or recurrent thrombosis on anticoagulation, prior to emergency procedures, in patients with renal impairment, at the extremes of body weight and suspected overdose [10,20]. 

The lack of routine available reversal agents remains a concern for the prescribers. While idarucizumab has now been approved and andexanet alfa is in the final phases of development, these are not readily available in most hospital and are likely to be at substantial financial cost. Hence, careful consideration of the use of these agents in individuals with high risk of bleeding continues to be warranted. 

While DOAC do not require monitoring of anticoagulation effect, it remains important that renal and liver function is monitor regularly, with the European Society of Cardiology suggesting that monitoring occurs every three to six months. It is particularly pertinent in the elderly population, where renal function in the elderly is often unstable and affected by concurrent illnesses and hospitalizations [33,34,35]. Additionally, renal function in older age is often overestimated (e.g., in the presence of malnutrition and sarcopenia), as serum urea, creatinine and eGFR may be misleadingly normal in such circumstances. Creatinine clearance (CrCl), preferably measured by the Cockcroft method, is therefore the preferred method of measuring renal function and should be estimated prior to commencing NOACS [34].

Compliance is another important issue given the short half-life of the DOAC, which means that any missed doses will lead to a period without effective anticoagulation. This is particularly important since WHO reports that 50% of patients with chronic illness do not comply with long-term medication use [36]. Given that adherence rates are generally higher in clinical trials (often above 80%) [37], it is important to monitor the effects of compliance on thrombotic complications in real-world Phase IV registries such as the Global Anticoagulant Registry in the Field—Venous Thrombolic Event (GARFIELD-VTE) observational study (NCT02155491). 

Moreover, there remains a paucity of data supporting the use of DOACs in certain clinical settings such as in patients with active malignancy, antiphospholipid syndrome, pregnancy and unusual sites of VTE (e.g., cerebral sinus thrombosis and portal vein thrombosis) although the authors note that there are ongoing clinical trials to address these issues [38]. A phase II trial evaluating the use of dabigatran in patients with mechanical heart valves was terminated prematurely due to increased rates of thromboembolic and bleeding complications and hence mechanical heart valve remains a contraindication [39]. Hence, it remains crucially important for us to consider these medical conditions prior to commencements of these agents. Similarly, DOACs should also be used with caution in vulnerable patients in extremes of age, extremes of weight, moderate to severe renal and/or liver disease and in the frail elderly [19]. The use of DOACs should be individualised given that no patient is quite the same and all relevant clinical details and laboratory investigations be taken into consideration.

## 4. Use in Frail Elderly Patients

The elderly population forms the largest group of patients requiring anticoagulation given the increasing prevalence of AF and VTE with age [1,2] but it is also this very same cohort of patients that are at increased risk of bleeding complications such as gastrointestinal bleeding as well as falls risk. Frailty is a complex but common geriatric syndrome with worse overall health outcomes and increased risks of concurrent medical comorbidities such as renal and hepatic dysfunction, hospitalisations, falls, cognitive impairment and mortality [9,35,40]. 

Twenty-five to thirty per cent of the patients enrolled in the RCTs are over 75 years of age [3,5] and a recent meta-analysis showed that both stroke risks and VTE-related complications were reduced in the DOACs group and there was no demonstrable statistical difference in bleeding complications between those who received DOACs *versus* warfarin [41]. However, chronological age itself is not a main indicator of frailty and these results cannot be automatically translated to the frail older population. Apart from multiple comorbidities and poorer health reserve, these patients are also more likely to be at risk of falls, malnutrition, polypharmacy and renal impairment [40,42,43,44]. Hence, without clear clinical trial or “real-world” experience of the use of DOAC in the frail elderly population, careful consideration of each individual comorbidities, thrombosis and bleeding risk is required before commencing DOACS.

**Figure 1:**
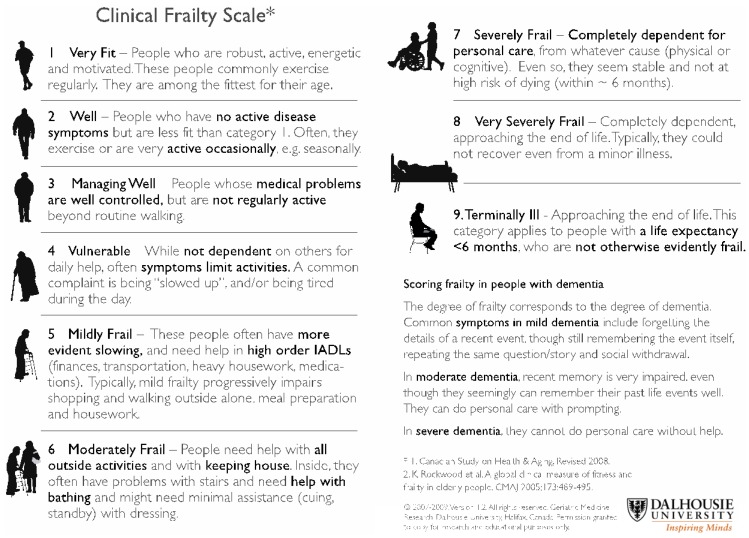
Clinical Frailty Scale (adapted from Rockwood *et al.* [31]).

**Figure 2:**
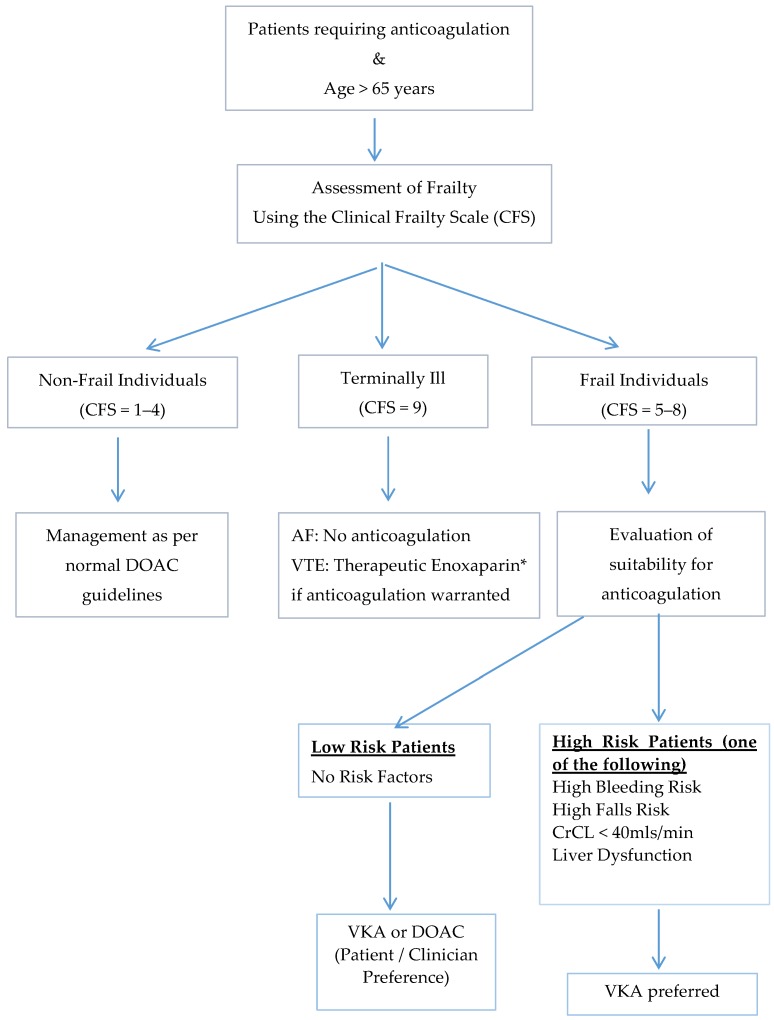
Proposed algorithm for the management of frail older adults requiring anticoagulation using the Clinical Frailty Score [35,45].

We recently published a review addressing the use of DOACs in the geriatric population in which an algorithm using the Clinical Frailty Score (CFS) to risk-stratify the patients is proposed (Figure 1 and Figure 2) [35,45]. Importantly, the first consideration in the geriatric population is the suitability of the use of any anticoagulant in these patients. Only after careful consideration of the risk-benefit of anticoagulation in each of these individuals, and discussion with the patient and caregiver, should any anticoagulation be prescribed. The CFS score (ranging from 1 (fit/robust) to 9 (terminally ill)) allows the identification of frail individuals and may be useful in determining suitability for DOACs. We have suggested that in the frail individuals (CFS score ≥5) with other significant risk factors such as high bleeding and falls risk, that VKA may be preferable to DOACs particularly in the setting of lack of drug measurement capacity and readily available reversal agents.

Moreover, it is important to realise frailty and medical issues in the elderly does not remain in stagnancy, and can change significantly. Particularly, the renal function can be quite sensitive to medical illnesses and use of concurrent medications [46] and hence, routine assessment of renal function is important. It is also important to remember that the estimated glomerular filtration rate (eGFR) is not accurate in many elderly patients and creatinine clearance is the preferred method of measuring renal function [44]. We suggest regular three to six monthly follow-up of renal and hepatic function, bleeding risk and frailty assessment in older patients on DOACs.

## 5. Approach to Decision-Making

As the number of patients on DOACs increases, this will translate to an increase in encounters with such patients by the dental surgeons who face making the decisions on the safety of dental procedures while they are on DOACs. However, there is a paucity of clinical trials and established evidence in the literature regarding the management of dental patients taking DOACs.

Crucial to any assessment of bleeding risks in patients on anticoagulants is the careful consideration of the bleeding risks associated with the procedure and the thromboembolic risks associated with the cessation of anticoagulation [11,47,48]. A detailed clinical history including bleeding history and concomitant medications, as well as other potential medical comorbidities that may compromise the renal and liver function, is important. This needs to be balanced with the bleeding risks associated with the specific dental procedure. We acknowledge that this assessment may not be straight forward, and further discussion with the patient’s prescribing physician or the haematologist may be warranted. 

The risks of bleeding due to dental procedures while on oral anticoagulants are predominantly studied in those on VKA and previous systematic review and meta-analysis have shown that perioperative continuation of warfarin with patients’ usual doses was not associated with an increased risk of bleeding [49,50,51]. An extrapolation of these results would suggest that in the absence of additional risks that impair haemostasis further, it may not be necessary to discontinue the use of DOACs especially in the minor procedures such as simple extractions, periodontal surgery and abscess incision although the procedures should ideally be performed at least 12 hours after the last dosing [11,34,47,52]. The UC Davis Health System Anticoagulation Services has recently released their recommendations for anticoagulation according to the various types of dental procedures as seen in Table 2 [13].

Local measures such as local pressure, site packing with oxidized cellulose or collagen sponges and additional suturing should be taken to minimize the bleeding during and after the dental procedures [13,50,51,53]. The procedures should be carried under local anaesthetic containing vasoconstrictor where appropriate and mouth rinses such as 5% tranexamic acid mouthwash can be given to the patients post procedure [12,50,51].

As opposed to warfarin, which may require the interruption of anticoagulation for several days prior to the dental procedures, patients on DOACs without additional risks of impaired haemostasis such as older age and impaired renal function, may only need to withhold their doses for a day due to the shorter half-life of the DOACs. Furthermore, due to the rapid onset of these DOACs, the patients also will achieve therapeutic anticoagulation more rapidly compared to warfarin without having to use parenteral “bridging” anticoagulants and minimizing the time off therapeutic anticoagulation and further reducing the thromboembolic risk due to interruption of anticoagulation. However, if it is decided that there is an appreciable bleeding risk associated with the dental procedure which outweighs the risk of thromboembolic event, Table 3 summarises the current recommendations of preoperative interruption of DOACs [19,54]. Generally, in procedural settings, anticoagulation is stopped between 24–72 h prior and should be restarted as soon as clinically appropriate. 

The patient should be instructed verbally and provided in writing about the usual postprocedural course and the measures to be taken in the event of bleeding. Non-steroidal anti-inflammatory drugs should not be prescribed for dental pain as they may impair haemostasis further.

## 6. Conclusions

The use of DOACs will continue to grow including in the elderly population. Frail older adults, however, are a unique population and we suggest a more conservative approach in this group, including careful evaluation of comorbidities, cognitive functional status as well as bleeding and falls risk. On the basis of limited evidence for dental procedures in particular, general recommendations appear to be similar to those of VKA, which is that most patients receiving DOACs do not require a change to their anticoagulation. However, in patients whom the bleeding risks outweigh the thromboembolic risk, temporary interruption of anticoagulation may be considered, in consultation with the prescribing physician or haematologist. Further studies are required to further validate these recommendations.

## Figures and Tables

**Table 1:** Pharmacologic properties of the DOACs (Adapted from Bauer *et al.*, Tran *et al.* and Dale *et al.* [18,19,20]).

	Dabigatran	Rivaroxaban	Apixaban
Mechanism of action	Direct thrombin inhibitor	Direct factor Xa inhibitor	Direct factor Xa inhibitor
Time to peak plasma concentration (in healthy adults)	2 h	2.5–4 h	1–3 h
Half-life (in healthy adults)	12–17 h	7–13 h	8–15 h
Elimination	Renal 80%, hepatic 20%	Renal 33%, renal metabolite 33%, hepatic 33%	Renal 25%, hepatic 75%
Indication	Stroke prevention in non-valvular AF	Stroke prevention in non-valvular AF VTE prophylaxis and treatment	Stroke prevention in non-valvular AF VTE prophylaxis and treatment
Dosing regimen	Twice daily	Once daily	Twice daily
Potential drug interactions	Potent P-glycoprotein (P-gp) inhibitors and P-gp inducers	Strong dual CYP 3A4 and P-gp inhibitors/inducers	Strong dual CYP 3A4 and P-gp inhibitors/inducers
Recommended laboratory tests:			
Significant anticoagulant effect unlikely	APTT is normal, Thrombin time (TT) is more sensitive than APTT and a normal TT results suggests low dabigatran level or absent drug	PT normal	Normal APTT and PT cannot be used to exclude anticoagulant effect.
Anticoagulant effect present (screening test)	TT prolonged; APTT prolonged	PT normal/prolonged	PT prolonged—apixaban likely present in excess. PT is only weakly sensitive to apixaban with inter-reagent variability and a normal PT does not rule out the presence of anticoagulant effect
Drug effect likely (confirmatory tests)	Dilute thrombin clotting time assay (HEMOCLOT) prolonged Ecarin clot time (ECA) Anti FIIa (chromogenic assay)	Modified specific anti-Xa positive	Modified specific anti-Xa positive
Antidote	Idarucuzimab [21] (approved by US FDA on 16 October 2015)	Andexanet alfa [22] (Phase III trial)	Andexanet alfa [22] (Phase III trial)

**Table 2:** Dental procedures according to bleeding risks and peri-procedural recommendations as made by the UC Davis Health System Anticoagulation Services [13].

Dental Procedure	Presumed Bleeding Risk	Peri-Procedural Recommendations
Supragingival scaling Simple restorations Local anaesthetic injections	Low	Continue therapeutic anticoagulation
Subgingival scaling Subgingival preparation restoration Standard root canal Simple extractions Regional anaesthetic injections	Moderate	Continue therapeutic anticoagulation
Extensive surgery Apicoectomy (root removal) Alevolar surgery (bone removal)	High	Consider reducing anticoagulation

**Table 3:** Preoperative interruption of DOACs (Adapted from Tran [19], van Rys [54]). * Neither rivaroxaban nor dabigatran should be used in the presence of severe renal impairment (CrCl < 30 mL/min). Apixaban should be avoided in patients with CrCl < 25 mL/min.

Drug	Renal Function	Low Bleeding Risk Surgery	High Bleeding Risk Surgery
Dabigatran	CrCl ≥ 50 mL/min	Last dose: 24 h before surgery	Last dose: 48–72 h before surgery
	CrCl 30–49 mL/min	Last dose: 48–72 h before surgery	Last dose: 96 h before surgery
	CrCl < 30 mL/min*	Last dose: 48–120 h before surgery	Last dose: ≥ 120 h before surgery
Rivaroxaban/Apixaban	CrCl ≥ 50 mL/min	Last dose: 24 h before surgery	Last dose: 48–72 h before surgery
	CrCl 30–49 mL/min	Last dose: 48 h before surgery	Last dose: 72 h before surgery
	CrCl < 30 mL/min*	Last dose: 48 h before surgery	Last dose: 72 h before surgery
